# Prevalence and Risk Factors of Hypertension, Diabetes, and Dyslipidemia among Adults in Northwest China

**DOI:** 10.1155/2021/5528007

**Published:** 2021-04-09

**Authors:** Lin Qiu, Weihua Wang, Rina Sa, Feng Liu

**Affiliations:** Shaanxi Provincial Center for Disease Control and Prevention, No. 3 Jiandong Street, Xi'an, China

## Abstract

**Objective:**

To understand the prevalence and its risk factors of hypertension, diabetes mellitus, and dyslipidemia among adults aged over 18 years in Northwest China and provide data for the prevention and control of chronic diseases in Northwest China.

**Methods:**

Three waves of survey on chronic diseases and nutrition monitoring with multistage stratified cluster random sampling were conducted in 10 counties of Northwest China in 2013, 2015, and 2018, respectively. Personal information, socioeconomic status (SES), and behavioral risk factors (cigarettes smoking, alcohol consumption, diets, and physical activity) were collected by face-to-face interview. Height, weight, and blood pressure were measured, and blood glucose and serum lipid were tested. Prevalence of hypertension, diabetes, and dyslipidemia of the three waves was estimated, and multivariate logistic regression was used to analyze their risk factors.

**Results:**

The prevalence of hypertension, diabetes, and dyslipidemia was 41.59%, 11.16%, and 32.48%, respectively. Their standardized prevalence was 29.31%, 7.94%, and 31.54%. Univariate analysis showed that the prevalence of hypertension, diabetes, and dyslipidemia was significantly different among educational levels, marital status, occupation, smoking, drinking, central obesity, and BMI classification (*P* < 0.05). Multivariate logistic regression analysis showed that factors like male gender, central obesity, overweight, and obesity were associated with hypertension, diabetes, and dyslipidemia (*P* < 0.05). High BMI index is one of the risk factors of the three diseases. The odds ratio (OR) of general overweight associated with hypertension, diabetes, and dyslipidemia was 1.663, 1.206, and 1.579 compared to normal body weight, respectively. And that was 3.094, 1.565, and 2.285 for obesity. Age was one of the risk factors for hypertension and diabetes (*P* < 0.05). Age groups of 45–59 years and 60 years and above were more associated with hypertension than of 18–44 age group (OR_45-59 group_ = 2.777, OR_60 years and above_ = 6.948), similar to their association with diabetes (OR_45-59 group_ = 2.357, OR_60 years and above_ = 3.521). Daily smoking is associated with diabetes and dyslipidemia (OR _for diabetes_ = 1.217, OR _for dyslipidemia_ = 1.287) and alcohol drinking associated with hypertension and dyslipidemia (OR _for hypertension_ = 1.014, OR _for__dyslipidemia_ = 1.034). Hypertension, diabetes, and dyslipidemia were also associated with each other (*P* < 0.05). The number of participants with hypertension, diabetes, and dyslipidemia accounted for 2.33% of all the participants, 15.60% for participants with hypertension and dyslipidemia, 4.58% for hypertension and diabetes, and 3.57% for diabetes and dyslipidemia, respectively.

**Conclusion:**

Factors like male, smoking, drinking, central obesity, overweight, and obesity were associated with hypertension, diabetes, and dyslipidemia in northwest China. Interventions on these risk factors and coexistence of the three diseases may help improve public health in this area.

## 1. Introduction

The living standards of Chinese people have been greatly improved with the rise economy, and their lifestyle and diet patterns have also changed. Noncommunicable chronic diseases, such as cardiovascular and cerebrovascular diseases, have become the main threat to public health of China [1]. Some prospective studies and clinical trials have suggested that hypertension, diabetes, and dyslipidemia are major risk factors for cardiovascular and cerebrovascular diseases [2]. Northwest China is dry and cold, especially in winter, and its economy is less developed. Health literacy of local residents was relatively weak. All resulted in cardiovascular and cerebrovascular diseases becoming the main health treat [3]. This study aimed to have a clue on the prevalence of hypertension, diabetes, and dyslipidemia among population over 18 years old in Northwest China with three cross-sectional surveys from 2013 to 2018. We also investigated the possible risk factors of hypertension, diabetes, and dyslipidemia to provide information on prevention and control chronic diseases locally.

## 2. Methods

### 2.1. Study Population

According to the *work plan for monitoring chronic diseases and nutrition of Chinese residents*, we carried out survey of chronic diseases and nutrition monitoring among adult in 2013, 2015, and 2018, in 10 counties and districts (Lianhu, Meixian, Chencang, Jingyang, Huayin, Huangling, Baota, Lueyang, Xunyang, and Shangzhou) in northwest China. The respondents were residents aged 18 or older who had lived in the monitored area for 6 months or more during the 12 months prior to the survey. Before the investigation, the subjects in the sampled area should be thoroughly investigated, and then the subjects were recruited by means of community/village committee broadcast publicity and village doctors' mobilization, etc. The investigation should be carried out in a centralized way.

### 2.2. Sample Size Calculation

The three waves of survey were cross-sectional. The sample size of each was calculated by stratified method. China divided into 31 layers based on its provincial level, multiplied by layers of urban and rural area, equaled to a total of 62 layers. The sample size is calculated as follows:

(1) $$\begin{array}{c} N=\text{deff}\frac{u^{2}p\left(1-p\right)}{d^{2}}, \end{array}$$

where *u* = 1.96, *р* is the prevalence of diabetes from the last year (9.7% in 2013 and 2015, and 10.4% in 2018), design efficacy (deff) is 3, the relative error (*r*) = 20%, and *d* = 20% × the prevalence of diabetes. According to the value of parameter and considering 10% of no-response rate, a total of 302 counties in China were selected to be the sample site, and 10 counties in Shaanxi Province in Northwest China were selected. In total, 18501 participants were enrolled in the three waves.

### 2.3. Sampling

We used a multistage cluster random sampling method in the three surveys. In the first stage, 3 townships/streets were randomly selected from each county. In the second stage, two villages were randomly selected from each selected township/street. In the third stage, one group of villager/resident was randomly selected from each selected village. In the fourth stage, 45 households were selected from each selected villagers residents group. All the residents aged 18 or above in the selected households were invited to the survey.

### 2.4. Measurements and Definition

#### 2.4.1. Study Variables and Measures

Face-to-face interview with questionnaires, physical examination, and laboratory test were adopted in the three surveys and carried out by investigators who had received unified training and passed the examination. Three parts were included in the survey as follows: socioeconomic status (residency, age, gender, nationality, education level, marital status, occupation, and family annual income), behavioral risk factors (smoking, drinking, diet, and physical activity), and biological risk factors (waist circumference, weight, height, blood pressure, blood glucose, and blood lipid). The electronic sphygmomanometer and measurement method were used to obtain the blood pressure data of the subjects. The Omron HBP-1300 electronic sphygmomanometer was used for blood pressure measurement. The blood pressure was measured for 3 times in a quiet environment (each interval was 1 min), and the mean value of the next 2 times was taken as the individual blood pressure value, accurate to 1 mm Hg. 10 ml of fasting venous blood was collected to detect fasting blood glucose, total cholesterol, triglyceride, high-density lipoprotein cholesterol, and low-density lipoprotein cholesterol; 75 g of anhydrous glucose was given orally to the subjects without diabetes history, and 2 ml of venous blood was collected 2 hours after taking glucose . Plasma blood glucose was determined by the hexokinase method or glucose oxidase method; total cholesterol was determined by the cholesterol oxidase amino-antipyrlophenol method (CHOD-PAP), triglyceride was determined by the glycerin phosphate oxidase 4-chloric acid method, and high-density lipoprotein cholesterol and low-density lipoprotein cholesterol were determined by the homogeneous enzyme colorimetry.

#### 2.4.2. Exposure and Outcome

Insufficient consumption of vegetables and fruits was defined as average daily intake <400 g. Excessive intake of red meat was defined as average daily intake >100 g. The average daily salt intake >6 g was defined as excessive salt intake [4]. Central obesity referred to male waist circumference ≥85 cm or female waist circumference ≥80 cm. BMI <18.5 kg/m^2^ is low body weight, 18.5 kg/m^2^ ≤ BMI < 24.0 kg/m^2^ is normal weight, 24.0 kg/m^2^ ≤ BMI < 28.0 kg/m^2^ is overweight, and BMI ≥ 28.0 kg/m^2^ is obesity [5]. Hypertensive patients: according to the Chinese guidelines for prevention and treatment of hypertension (revised version in 2018) [6], hypertension is defined as SBP ≥ 140 mm Hg and/or DBP ≥90 mm Hg or those who had been diagnosed with hypertension in hospitals and took antihypertensive drugs in recent 2 weeks [6]. Diabetics were those with fasting blood glucose (FBG) ≥7.0 mmol/L and/or 2-hour oral glucose tolerance test blood glucose (OGTT) ≥11.1 mmol/l or those who have been diagnosed with diabetes by hospitals [7]. Total cholesterol ≥6.22 mmol/L is hypercholesterolemia; HDL-C < 1.04 mmol/L is low-density lipoprotein cholesterol; low-density lipoprotein cholesterol ≥4.14 mmol/L is high-density lipoprotein cholesterol; and triglyceride ≥2.26 mmol/L is hypertriglyceridemia. Those who meet any of the above are defined as dyslipidemia [8].

### 2.5. Statistical Analysis

The continuous data were described by $\bar{\chi }$ ± *s*. The categorized data were described by frequency and proportion and compared by the *χ*^2^ test. Age-standardized prevalence of hypertension, diabetes, and dyslipidemia in the study was calculated using the 6th National Census of China in 2010. The multivariate logistic regression model was used to examine the association of hypertension, diabetes, dyslipidemia, and the related risk factors. Model I was constructed to explore their association. If *р* value < 0.20, the risk factors were involved in the model II to determine their associations. We set *α* = 0.05 to judge the statistical significance. SPSS 25.0 software was used for statistical analysis in the study. Variables and their values are shown in Table 1. The logistic regression model is as follows:

(2) $$\begin{array}{c} \text{Logit }\left(P\right)=\ln\left[\beta_{0}+\beta_{1}X_{0}+\cdots +\beta_{m}X_{m}\right]. \end{array}$$

where *β*_0_ is a constant term or intercept and *β*_1_, *β*_2_,…,*β*_*m*_ are for the logistic regression model of regression coefficient, according to the sample data to estimate the constant term and coefficient of regression of logistic regression model, and then we describe and analyze the relationship between the response variable and the independent variable and calculate the probability of the results under the characteristic conditions.

## 3. Results

### 3.1. Characteristics of the Participants

The total population of the 10 counties is 4023603. A total of 18501 participants at 18 years old or above in northwest China were investigated in the survey from 2013 to 2018. The average age was (51.51 ± 14.01) years old. Among all the participants, 8610 (46.54%) were males and 9891 (53.46%) were females. 7337 (39.66%) were from urban areas, and 11164 (60.34%) were from rural areas. The number, (prevalence and age-standardized prevalence) of hypertension, diabetes, and dyslipidemia patients was 7656 (41.59%, 29.31%), 1340 (11.16%, 7.94%), and 5887 (32.48%, 31.54%), respectively. The prevalence of hypertension, diabetes, and dyslipidemia was significantly different in the education level, marital status, occupation, smoking, drinking, central obesity, and BMI groups (*P* < 0.05).  Table 2.

### 3.2. Cluster Analysis of Hypertension, Diabetes, and Dyslipidemia

**Figure 1:**
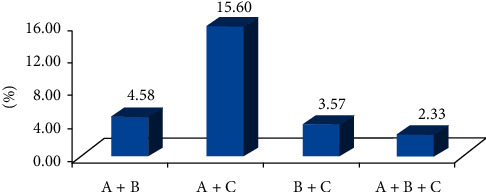
The overlap of hypertension, diabetes, and dyslipidemia in Northwest China. *Note.* A is hypertension, B is diabetes, and C is dyslipidemia. The percentages shown in each part of the figure are the proportion of the diseases in the population.

The results showed that the number of people with three diseases accounted for 2.33%, and the number of people with hypertension and diabetes, hypertension and dyslipidemia, and diabetes and dyslipidemia accounted for 4.58%, 15.60%, and 3.57%, respectively (Figure 1).

### 3.3. Risk Factor Analysis

#### 3.3.1. Risk Factors of Hypertension

The results in model I showed that there were no association between family average annual income, smoking, daily average red meat intake, and hypertension (*P* > 0.200). The rest of the risk factors in Table 1 are included in model II, the results showed that gender, age, alcohol consumption, central obesity, overweight and obesity, insufficient daily intake of vegetables and fruits, diabetes, and dyslipidemia were associated with hypertension. Those who were 45–59 years old and 60 years old and above were 2.78 times (95% CI 2.475–3.116) and 6.948 times (95% CI 6.107–7.905) prone to have hypertension than those at 18–44 years old, respectively. Overweight and obesity were 1.66 times (95% CI 1.489–1.858) and 3.094 times (95% CI) to have hypertension than those with normal weight, respectively. Participants with diabetes were 1.82 times (95% CI 1.586–2.085) to have hypertension than those without. And that was 1.247 times (95% CI 1.133–1.371) in participants with dyslipidemia. Participants in rural areas, graduated from middle school, junior college or above, unmarried or married and cohabitation, and low weight were less likely to having hypertension (Table 3).

#### 3.3.2. Risk Factors of Diabetes

The results in model II showed that gender, age, daily smoking, central obesity, overweight and obesity, hypertension, and dyslipidemia were associated with diabetes. Those who were 45–59 years old and 60 years old and above were 2.357 times (95% CI 1.952–2.847) and 3.521 times (95% CI 2.894–4.285) prone to have risk of diabetes than those who were 18–44 years old, respectively. Those who were overweight and obesity were 1.206 times (95% CI 1.028–1.415) and 1.565 times (95% CI 1.284–1.907) to have the risk of diabetes than those with normal weight, respectively. Participants with hypertension or dyslipidemia were 1.761 times (95% CI 1.546–2.006) and 1.934 times (95% CI 1.708–2.189) to get diabetes than those without them, respectively. Farmers and those who were living in rural areas were less likely to get diabetes (Table 4).

#### 3.3.3. Risk Factors of Dyslipidemia

The results in model II showed that gender, education level, daily smoking, drinking, central obesity, overweight and obesity, hypertension, and diabetes were associated with dyslipidemia. Overweight and obesity were 1.579 times (95% CI 1.420–1.755) and 2.285 times (95% CI 1.986–2.628) prone to have the risk of dyslipidemia than those with normal weight, respectively. Those with hypertension were 1.242 times (95% CI 1.134–1.360) prone to have the risk of dyslipidemia than those without it. And that was 1.924 times (95% CI 1.699–2.179) for participants with diabetes. Low body weight was less likely to get dyslipidemia (Table 5).

## 4. Discussion

In the past 30 years, the increase of incidence and prevalence of hypertension, diabetes, and dyslipidemia in China has resulted in the growth of burden of these three diseases [9]. With the rapid economic development, changes in people's lifestyle and eating habits and the accelerated aging of the population, cardiovascular, and cerebrovascular diseases have become the first mortality among Chinese residents currently. It is well documented that hypertension, diabetes, and dyslipidemia are significant risk factors for cardiovascular and cerebrovascular diseases [9, 10]. It is of great significance to estimate the prevalence of these three diseases in Western China and explore their risk factors for the prevention and control of chronic diseases in Northwest China.

According to this study, the prevalence of hypertension in northwest China (41.59%, age-standardized 29.31%) was higher than that of overall China (23.5%) in 2015. And, the prevalence of diabetes (11.16%, age-standardized 7.94%) and dyslipidemia (32.48%, age-standardized 31.54%) was lower than that of overall China in 2013 and in 2015, respectively. Due to diversities of regions, lifestyles, and eating habits, the disease prevalence in Northwest China is different from the national average. The prevalence of diabetes and dyslipidemia in rural areas was higher than that in urban areas. The possible reasons might be the insufficient distribution of health resources in rural areas in northwest China, the scattered residence in mountains with inconvenient transportation, and the relatively low education level of the local residents. They all resulted to the rural residents having less knowledge on the prevention and treatment of chronic diseases and did not pay attention to information on health [11]. The prevalence of hypertension, diabetes, and dyslipidemia among residents with low education background (middle school and below) was higher than that of college students and above. This may be because the health awareness and healthy lifestyle were related to the education level. The awareness is relatively weak, and healthy lifestyles were not easy to be developed among people with low education level [10, 11]. The prevalence of hypertension and diabetes in middle-aged and elderly people was higher than that in the young people. On the one hand, it may be vascular sclerosis and decreased elasticity with the increase of age [12]; on the other hand, the elderly people exposed to more health examination than young people because of having many diseases enhanced the detection rate.

The study showed that men were more likely than women to have hypertension, diabetes, and dyslipidemia, which was related to unhealthy lifestyle and diets among men. The majority of men smoked and drank and liked to have greasy food, and they had abdominal obesity [13]. The risk to have hypertension and diabetes was associated with older age, which may be related to the fact that the middle-aged and elderly people were more vulnerable to multiple chronic diseases including hypertension, diabetes, and their complications due to the decline of physical function [14]. Central obesity, overweight, and obesity were risk factors for hypertension, diabetes, and dyslipidemia. These three diseases were also related to higher BMI. This is consistent with the studies in other areas of China [11–13]. It is suggested that the main interventions to prevent the occurrence of chronic diseases are to advocate residents to adhere to regular physical exercise, to watch weight changes, and to carry out health education on diet with low salt, low fat, and low oil. This way will help residents to develop a healthy lifestyle, improve the lipid metabolism, and maintain a healthy body weight [14]. Daily smoking is a risk factor for diabetes and dyslipidemia. Drinking is a risk factor for hypertension and dyslipidemia. It demonstrated in this study that those who are male, daily smoking, drinking, central obesity, overweight, and obesity are the high-risk population in prevention and control of hypertension, diabetes. and dyslipidemia in western China.

The World Health Organization (WHO) defined 2 or more chronic diseases in the same patient for more than 6 months as comorbidity in 2008, which increased the risk of death, hospitalization, disability, depression, and economic expenditure [15, 16]. Research showed that hypertension, diabetes, and other diseases were often regarded as the key diseases of comorbidity [17]. The study found that 2.33% of the participants had hypertension, diabetes, and dyslipidemia at the same time. The comorbidity of hypertension and dyslipidemia (15.60%) was much higher than that of hypertension and diabetes (4.58%) or diabetes and dyslipidemia (3.57%). This is consistent with that of the study in Nanchong in China, which may be related to the prevalence of hypertension being higher than that of diabetes or dyslipidemia. The study showed that hypertension, diabetes, and dyslipidemia are linked to each other, suggesting that interactions of these three diseases on population health should be noticed. Hypertension affected the blood lipid metabolism and resulted in dyslipidemia further. Meanwhile, dyslipidemia increased the variability of blood pressure. The blood lipid level and blood pressure were associated and affected each other [18]. Insufficient insulin secretion stimulated fatty acids released in the human body. Dyslipidemia and diabetes could largely increase the risk of cardiovascular disease and mortality [19]. Therefore, it is necessary to control and prevent the risk factors of these three diseases in order to reduce the risk of cardiovascular and cerebrovascular events.

In conclusion, chronic diseases affected the health of the population in Northwest China. To reduce the prevalence and mortality of chronic diseases such as cardiovascular and cerebrovascular diseases, it is necessary to monitor metabolic indicators such as blood pressure, blood lipid, and blood glucose and take measures to prevent and control the hypertension, diabetes, dyslipidemia, and obesity [18, 19]. Community management of chronic disease is the main mode in Northwest China. The prevention and control system of the state, province, city, county, and township/community can be used to carry out interventions on prevention, control, and education based on the characteristics of different groups of people, so as to promote the healthy lifestyle and reduce the occurrence of risk factors [20]. First, primary care health service institutions should combine with the *national basic public health service* to strengthen the management of residents' blood pressure, blood sugar, and blood lipid and to guide the rational drug use. Second, it is suggested that the health institutions should carry out health education on knowledge of chronic disease prevention and control and advocate residents to promote physical activity and keep balanced diet. Third, we could find the key population by early screening to intervene the main risk factors and further to control the occurrence and development of chronic diseases by taking comprehensive measures. Fourth, it is suggested to establish cooperation between community health service centers/township health centers and higher-level general hospitals to give guidance and treatment to high-risk groups [21].

The prevalence, comorbidity, and risk factors of hypertension, diabetes, and dyslipidemia from this study could provide basic information for chronic disease prevention and control in northwest China. However, there are several limitations in this study. First, the relatively high proportion of elderly participants might lead to a high prevalence of the three diseases, for age is a risk factor for diseases such as hypertension and diabetes; second, the correlation between physical activity and the three diseases was not considered in this study, which should be studied further.

## Figures and Tables

**Table 1:** Values of variables in multivariate logistic regression model.

Variables	Values
Sex	0 = female; 1 = male
Residency	0 = urban areas; 1 = rural areas
Age (year)	0 = 18–44 years old; 1 = 45–59 years old; 2 = 60 years old or above
Ethnicity	0 = others; 1 = Han
Education level	0 = primary school or below; 1 = middle school; 2 = college or above
Marital status	0 = separated/divorced/widowed; 1 = single; 2 = married or cohabit
Occupation	0 = others; 1 = farmers
Family income (RMB)	0 = <5000; 1 = 5000–9999; 2 = 10000–19999; 3 =≥20000
Cigarette smoking	0 = never; 1 = daily smoking; 2 = non-daily smoking; 3 = ex-smoking
Alcohol drinking	0 = never; 1 = drinking in the past 12 month; 2 = drinking in the past 1 month
Daily consumption of vegetables and fruits (g)	0 = <400, 1 = >400
Daily salt intake (g)	0 = <6, 1 =>6
Daily red meat intake (g)	0 = <100, 1=>100
BMI groups	0 = normal body weight; 1 = underweight; 2 = overweight; 3 = obesity
Central obesity	0 = no; 1 = yes
Hypertension	0 = no; 1 = yes
Diabetes	0 = no; 1 = yes
Dyslipidemia	0 = no; 1 = yes

**Table 2:** Baseline of the participants in northwest China.

Variables	Hypertension			Diabetes			Dyslipidemia		
	Yes	No	*P*	Yes	No	*P*	Yes	No	*P*
Overall	7656 (41.59)	10752 (58.41)		1340 (11.16)	10668 (88.84)		5887 (32.48)	12239 (67.52)	
*Residency*									
Urban	3066 (40.05)	4252 (39.55)	0.494	650 (48.51)	4097 (38.4)	＜0.001	2455 (41.7)	4756 (38.86)	＜0.001
Rural	4590 (59.95)	6500 (60.45)		690 (51.49)	6571 (61.6)		3432 (58.30)	7483 (61.14)	

*Sex*									
Female	4131 (53.96)	5718 (53.18)	0.298	688 (51.34)	5872 (55.04)	0.010	2780 (47.22)	6932 (56.64)	＜0.001
Male	3525 (46.04)	5034 (46.82)		652 (48.66)	4796 (44.96)		3107 (52.78)	5307 (43.36)	

*Ethnicity*									
Others	57 (0.74)	105 (0.98)	0.097	22 (1.64)	120 (1.13)	0.099	51 (0.87)	108 (0.88)	0.915
Han	7595 (99.26)	10641 (99.02)		1317 (98.36)	10539 (98.87)		5833 (99.13)	12128 (99.12)	

*Education level*									
Primary school or below	4081 (53.35)	4052 (37.72)	＜0.001	617 (46.08)	4515 (42.38)	0.031	2392 (40.66)	5613 (45.89)	＜0.001
Middle school	3303 (43.18)	5805 (54.04)		629 (46.98)	5394 (50.63)		3086 (52.46)	5892 (48.17)	
College or above	266 (3.48)	886 (8.25)		93 (6.95)	745 (6.99)		405 (6.88)	727 (5.94)	

*Marital status*									
Separated/divorced/widowed	714 (9.33)	498 (4.64)	＜0.001	131 (9.78)	775 (7.27)	＜0.001	355 (6.03)	833 (6.81)	＜0.001
Single	147 (1.92)	711 (6.62)		26 (1.94)	559 (5.25)		265 (4.5)	576 (4.71)	
Married or cohabit	6791 (88.75)	9535 (88.75)		1182 (88.27)	9323 (87.48)		5264 (89.46)	10825 (88.48)	

*Occupation*									
Others	3384 (44.23)	5249 (48.85)	＜0.001	749 (55.94)	5281 (49.55)	＜0.001	2878 (48.92)	5621 (45.94)	＜0.001
Farmer	4267 (55.77)	5496 (51.15)		590 (44.06)	5376 (50.45)		3005 (51.08)	6614 (54.06)	
Age (year)	57.92 ± 11.79	47.02 ± 13.66		58.18 ± 12.00	50.68 ± 14.16		51.81 ± 13.78	51.45 ± 14.05	
18–44	1020 (13.32)	4652 (43.27)	＜0.001	175 (13.06)	3589 (33.64)	＜0.001	1773 (30.12)	3797 (31.02)	0.464
45–59	3137 (40.97)	4195 (39.02)		550 (41.04)	4182 (39.2)		2369 (40.24)	4864 (39.74)	
≥60	3499 (45.70)	1905 (17.72)		615 (45.9)	2897 (27.16)		1745 (29.64)	3578 (29.23)	

*Family income (RMB)*									
<5000	358 (8.83)	290 (5.66)	＜0.001	30 (6.32)	247 (6.38)	0.725	204 (6.74)	437 (7.31)	0.716
5000–9999	383 (9.45)	370 (7.22)		39 (8.21)	263 (6.79)		244 (8.06)	490 (8.19)	
10000–19999	726 (17.91)	801 (15.63)		68 (14.32)	566 (14.62)		516 (17.05)	985 (16.47)	
≥20000	2586 (63.80)	3664 (71.49)		338 (71.16)	2796 (72.21)		2062 (68.14)	4069 (68.03)	

*Cigarette smoking*									
Never	2753 (25.61)	1761 (23.00)	＜0.001	2597 (24.35)	273 (20.37)	＜0.001	2769 (22.62)	1664 (28.27)	＜0.001
Daily smoking	247 (2.30)	202 (2.64)		247 (2.32)	32 (2.39)		291 (2.38)	149 (2.53)	
Non-daily smoking	451 (4.20)	518 (6.77)		479 (4.49)	120 (8.96)		595 (4.86)	362 (6.15)	
Ex-smoking	7297 (67.89)	5175 (67.59)		7341 (68.84)	915 (68.28)		8584 (70.14)	3712 (63.05)	

*Alcohol drinking*									
Never	1805 (16.80)	1197 (15.63)	＜0.001	1492 (13.99)	166 (12.39)	0.028	1914 (15.64)	1045 (17.75)	＜0.001
Drinking in the past 12 months	1516 (14.11)	753 (9.84)		1512 (14.18)	165 (12.31)		1412 (11.54)	826 (14.03)	
Drinking in the past 1 month	7425 (69.10)	5706 (74.53)		7659 (71.83)	1009 (75.30)		8912 (72.82)	4016 (68.22)	

*Central obesity*									
No	2701 (35.47)	5777 (54.77)	＜0.001	362 (27.28)	5047 (47.61)	＜0.001	1800 (30.72)	6610 (54.34)	＜0.001
Yes	4913 (64.53)	4771 (45.23)		965 (72.72)	5554 (52.39)		4060 (69.28)	5554 (45.66)	

*BMI groups*									
Normal body weight	2858 (37.52)	5750 (54.52)	＜0.001	440 (33.16)	5227 (49.31)	＜0.001	1932 (32.97)	6613 (54.36)	＜0.001
Underweight	138 (1.81)	402 (3.81)		23 (1.73)	334 (3.15)		59 (1.01)	475 (3.9)	
Overweight	3084 (40.49)	3450 (32.71)		567 (42.73)	3735 (35.23)		2584 (44.1)	3900 (32.06)	
Obesity	1537 (20.18)	945 (8.96)		297 (22.38)	1305 (12.31)		1285 (21.93)	1178 (9.68)	

*Daily salt intake (g)*									
<6	2809 (38.46)	4195 (41.28)	＜0.001	548 (44.05)	4229 (43.05)	0.503	2363 (42.36)	4518 (38.88)	＜0.001
>6	4495 (61.54)	5967 (58.72)		696 (55.95)	5594 (56.95)		3215 (57.64)	7101 (61.12)	

*Daily consumption of vegetable and fruit (g)*									
>400	2972 (39.54)	5101 (47.99)	＜0.001	705 (52.61)	5710 (53.54)	0.519	2652 (45.65)	5307 (44)	0.038
<400	4545 (60.46)	5528 (52.01)		635 (47.39)	4954 (46.46)		3158 (54.35)	6755 (56)	

*Daily red meat intake (g)*									
<100	5846 (94.92)	8429 (93.20)	＜0.001	1240 (92.54)	9936 (93.17)	0.387	4490 (92.75)	9589 (94.38)	＜0.001
>100	313 (5.08)	615 (6.80)		100 (7.46)	728 (6.83)		351 (7.25)	571 (5.62)	

**Table 3:** Logistic regression analysis on risk factors of hypertension among adults from northwest China.

Variables	Model I		Model II	
	OR (95% CI)	*P*	OR (95% CI)	*P*
*Residency*				
Rural	0.836 (0.716–0.975)	0.023	0.906 (0.826–0.995)	0.039

*Sex*				
Male	1.260 (1.033–1.536)	0.023	1.106 (1.003–1.219)	0.042

*Ethnicity*				
Han	1.628 (0.917–2.890)	0.096	1.294 (0.843–1.985)	0.238

*Education level*				
Middle school	0.823 (0.712–0.952)	0.009	0.801 (0.729–0.880)	＜0.001
College or above	0.560 (0.393–0.799)	0.001	0.574 (0.461–0.714)	＜0.001

*Marital status*				
Single	0.546 (0.322–0.924)	0.024	0.557 (0.409–0.758)	＜0.001
Married or cohabit	0.709 (0.536–0.938)	0.016	0.811 (0.690–0.954)	0.011

*Occupation*				
Farmer	1.090 (0.941–1.264)	0.251		

*Age (year)*				
45–59	2.582 (2.141–3.113)	＜0.001	2.777 (2.475–3.116)	＜0.001
≥60	6.158 (5.013–7.565)	＜0.001	6.948 (6.107–7.905)	＜0.001

*Family income (RMB)*				
5000–9999	1.000 (0.696–1.435)	0.999		
10000–19999	1.186 (0.863–1.630)	0.292		
≥20000	1.080 (0.815–1.431)	0.591		

*Cigarette smoking*				
Daily smoking	0.944 (0.759–1.175)	0.606		
Non-daily smoking	1.269 (0.790–2.037)	0.325		
Ex-smoking	0.973 (0.702–1.349)	0.871		

*Alcohol drinking*				
Drinking in the past 12 months	0.752 (0.587–0.963)	0.347	0.780 (0.678–0.896)	0.833
Drinking in the past 1 month	0.912 (0.752–1.105)	0.024	1.014 (0.889–1.158)	＜0.001

*Central obesity*				
Yes	1.384 (1.168–1.639)	＜0.001	1.383 (1.239–1.544)	＜0.001

*BMI groups*				
Underweight	0.532 (0.322–0.876)	0.013	0.559 (0.414–0.753)	＜0.001
Overweight	1.597 (1.348–1.892)	＜0.001	1.663 (1.489–1.858)	＜0.001
Obesity	2.805 (2.207–3.566)	＜0.001	3.094 (2.653–3.609)	＜0.001

*Daily salt intake (g)*				
>6	1.161 (1.007–1.338)	0.040	1.050 (0.961–1.148)	0.282

*Daily consumption of vegetables and fruits (g)*				
<400	1.152 (1.005–1.322)	0.043	1.273 (1.167–1.389)	＜0.001

*Daily red meat intake (g)*				
>100	0.954 (0.664–1.371)	0.800		

*Diabetes*				
Yes	2.207 (1.754–2.777)	＜0.001	1.818 (1.586–2.085)	＜0.001

*Dyslipidemia*				
Yes	1.227 (1.057–1.423)	0.007	1.247 (1.133–1.371)	＜0.001

*Note.* In model I, hypertension is taken as dependent variable; residency, age, sex, ethnicity, education level, marital status, occupation, family income, smoking, drinking, central obesity, BMI groups, salt intake, vegetable and fruit consumption, red meat intake, diabetes, and dyslipidemia are taken as independent variables. In model II, we further excluded family average annual income, smoking, and daily red meat intake, as their *P* > 0.20 in model I.

**Table 4:** Logistic regression analysis on risk factors of diabetes among adults from northwest China.

Variables	Model I		Model II	
	OR (95% CI)	*P*	OR (95% CI)	*P*
*Residency*				
Rural	0.743 (0.589–0.938)	0.012	0.706 (0.621–0.803)	＜0.001

*Sex*				
Male	1.256 (0.939–1.679)	0.124	1.351 (1.148–1.588)	＜0.001

*Ethnicity*				
Han	0.927 (0.457–1.881)	0.833		

*Education level*				
Middle school	1.006 (0.804–1.260)	0.955		
College or above	1.355 (0.817–2.248)	0.239		

*Marital status*				
Single	0.488 (0.180–1.325)	0.159	0.685 (0.430–1.090)	0.110
Married or cohabit	0.893 (0.617–1.293)	0.549	0.950 (0.770–1.171)	0.628

*Occupation*				
Farmer	0.740 (0.591–0.926)	0.009	0.783 (0.689–0.891)	＜0.001

*Age (year)*				
45–59	2.723 (1.886–3.931)	＜0.001	2.357 (1.952–2.847)	＜0.001
≥60	4.075 (2.794–5.942)	＜0.001	3.521 (2.894–4.285)	＜0.001

*Family income (RMB)*				
5000–9999	1.310 (0.766–2.242)	0.324		
10000–19999	1.099 (0.676–1.786)	0.703		
≥20000	1.107 (0.719–1.704)	0.644		

*Cigarette smoking*				
Daily smoking	1.104 (0.720–1.694)	0.083	1.217 (0.946–1.566)	＜0.001
Non-daily smoking	0.940 (0.467–1.890)	0.862	0.895 (0.594–1.347)	0.594
Ex-smoking	0.749 (0.540–1.039)	0.649	0.715 (0.592–0.864)	0.126

*Alcohol drinking*				
Drinking in the past 12 months	0.938 (0.699–1.260)	0.672		
Drinking in the past 1 month	1.013 (0.700–1.464)	0.947		

*Central obesity*				
Yes	1.627 (1.226–2.159)	0.001	1.456 (1.234–1.717)	＜0.001
BMI groups underweight	0.919 (0.360–2.350)	0.860	1.151 (0.736–1.800)	0.538
Overweight	1.153 (0.884–1.504)	0.293	1.206 (1.028–1.415)	0.022
Obesity	1.411 (1.016–1.958)	0.040	1.565 (1.284–1.907)	＜0.001

*Daily salt intake (g)*				
>6	0.924 (0.744–1.147)	0.475		

*Daily consumption of vegetable and fruit (g)*				
<400	0.968 (0.786–1.193)	0.763		

*Daily red meat intake (g)*				
>100	1.262 (0.744–2.138)	0.388		

*Hypertension*				
Yes	2.206 (1.753–2.777)	＜0.001	1.761 (1.546–2.006)	＜0.001

*Dyslipidemia*				
Yes	2.010 (1.630–2.478)	＜0.001	1.934 (1.708–2.189)	＜0.001

*Note.* In model I, hypertension is taken as dependent variable; residency, age, sex, ethnicity, education level, marital status, occupation, family income, smoking, drinking, central obesity, BMI groups, salt intake, vegetable and fruit consumption, red meat intake, diabetes, and dyslipidemia are taken as independent variables. In model II, we further excluded ethnicity, education level, family income, drinking, salt intake, daily red meat intake, and red meat intake, as their *P* > 0.20 in model I.

**Table 5:** Logistic regression analysis on risk factors of dyslipidemia among adults from northwest China.

Variables	Model I		Model II	
	OR (95% CI)	*P*	OR (95% CI)	*P*
*Residency*				
Rural	0.927 (0.792–1.085)	0.343		

*Sex*				
Male	1.245 (1.018–1.522)	0.033	1.401 (1.245–1.576)	＜0.001

*Ethnicity*				
Han	0.621 (0.365–1.057)	0.079	1.031 (0.709–1.500)	0.872

*Education level*				
Middle school	1.166 (1.004–1.354)	0.044	1.174 (1.072–1.286)	0.001
College or above	1.096 (0.790–1.522)	0.583	1.377 (1.155–1.642)	＜0.001

*Marital status*				
Single	1.078 (0.658–1.766)	0.766		
Married or cohabit	1.047 (0.786–1.395)	0.753		

*Occupation*				
Farmer	1.022 (0.878–1.189)	0.777		

*Age (year)*				
45–59	0.814 (0.677–0.977)	0.027	0.910 (0.820–1.009)	0.075
≥60	0.799 (0.650–0.983)	0.034	0.917 (0.813–1.036)	0.164

*Family income (RMB)*				
5000–9999	0.874 (0.596–1.283)	0.493		
10000–19999	1.061 (0.762–1.478)	0.726		
≥20000	1.065 (0.793–1.430)	0.677		

*Cigarette smoking*				
Daily smoking	1.309 (1.048–1.635)	0.018	1.287 (1.130–1.467)	＜0.001
Non-daily smoking	0.873 (0.533–1.430)	0.590	0.907 (0.680–1.209)	0.505
Ex-smoking	1.169 (0.842–1.624)	0.350	0.988 (0.805–1.212)	0.906

*Alcohol drinking*				
Drinking in the past 12 months	0.855 (0.665–1.099)	0.007	1.034 (0.910–1.175)	0.041
Drinking in the past 1 month	0.764 (0.628–0.929)	0.222	0.874 (0.769–0.994)	0.611

*Central obesity*				
Yes	1.683 (1.410–2.010)	＜0.001	1.952 (1.754–2.172)	＜0.001
BMI groups underweight	0.347 (0.159–0.760)	0.008	0.565 (0.403–0.793)	0.001
Overweight	1.778 (1.497–2.113)	＜0.001	1.579 (1.420–1.755)	＜0.001
Obesity	2.940 (2.339–3.696)	＜0.001	2.285 (1.986–2.628)	＜0.001

*Daily salt intake (g)*				
>6	1.018 (0.880–1.177)	0.812		

*Daily consumption of vegetable and fruit (g)*				
<400	1.054 (0.917–1.213)	0.459		

*Daily red meat intake (g)*				
>100	0.698 (0.482–1.011)	0.057	1.097 (0.935–1.287)	0.257

*Hypertension*				
Yes	1.210 (1.042–1.404)	0.012	1.242 (1.134–1.360)	＜0.001

*Diabetes*				
Yes	1.997 (1.619–2.462)	＜0.001	1.924 (1.699–2.179)	＜0.001

*Note.* In model I, hypertension is taken as dependent variable; residency, age, sex, ethnicity, education level, marital status, occupation, family income, smoking, drinking, central obesity, BMI groups, salt intake, vegetable and fruit consumption, red meat intake, diabetes, and dyslipidemia are taken as independent variables. In model II, we further excluded residency, marital status, occupation, family income, salt intake, daily red meat intake, and red meat intake, as their *P* > 0.20 in model I.

## Data Availability

The data used to support the findings of this study are available from the corresponding author upon request.
